# An efficient and affordable laboratory method to produce and sustain high concentrations of microcystins by *Microcystis aeruginosa*

**DOI:** 10.1016/j.mex.2019.10.024

**Published:** 2019-10-31

**Authors:** René S. Shahmohamadloo, Xavier Ortiz Almirall, Claire Holeton, Richard Chong-Kit, David G. Poirier, Satyendra P. Bhavsar, Paul K. Sibley

**Affiliations:** a School of Environmental Sciences, University of Guelph, Guelph, Ontario, Canada; b Ministry of the Environment, Conservation and Parks, Toronto, Ontario, Canada; c School of Environmental Studies, Queen’s University, Kingston, Ontario, Canada; d Department of Physical & Environmental Sciences, University of Toronto, Toronto, Ontario, Canada

**Keywords:** Method for production of microcystins in Blue-Green-11 (BG-11) medium, Strain CPCC 300, Cyanobacteria, Cyanotoxins, Harmful algal blooms, Toxicology

## Abstract

*Microcystis aeruginosa* is a cosmopolitan cyanobacteria that continues to jeopardize freshwater ecosystem services by releasing the hepatotoxin microcystin, which can, in some cases, cause death to aquatic fauna and even humans. Currently, our abilities to understand the mechanisms of microcystin toxicology are limited by the lack of a method for producing high concentrations, which are central to large-scale and long-term research in natural systems. Here we present an efficient and affordable laboratory method to produce high concentrations of microcystins by a toxigenic strain of *M. aeruginosa*. Through batch culture studies, we yielded microcystins at concentrations that are environmentally relevant to freshwaters around the world (1–300 μg L^−1^), maintained these concentrations without resupplying fresh medium (further reducing costs), and utilized rate equations to model the relationship between the environmental conditions in the cultures and changes occurring within the *M. aeruginosa* cells. Our assessment suggests that steady production of microcystins depends on the availability of carbon throughout the experiment. Hence, we recommend the use of tissue culture treated flasks with a vented cap to ensure the production of microcystins is uninterrupted. This method demonstrates that microcystins can be produced in the laboratory at concentrations relevant to freshwater ecosystems.

• The method demonstrates *M. aeruginosa* CPCC 300 is a reliable strain of freshwater cyanobacteria that can yield microcystins at environmentally relevant concentrations.
• Validation showed *M. aeruginosa* CPCC 300 is resilient in carbon-limited situations and may respond to stress by shifting the ratio of microcystin congeners.
• Cell culture flasks with vented caps —filled no more than 50 % of the flask volume to allow for sufficient air exchange— are an excellent and cost-effective approach to maintaining cell growth and producing microcystins at a range between 300 to 1200 μg L^−1^.

## Graphical abstract

**Figure:**
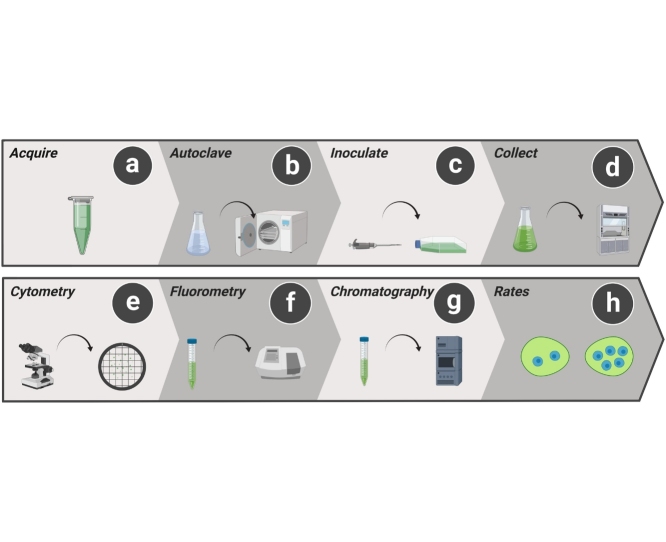

**Specification Table**

**Table:** 

Subject area:	Environmental Science
More specific subject area:	Microcystins, Harmful Algal Blooms, Freshwaters
Method name:	Method for production of microcystins in Blue-Green-11 (BG-11) medium
Name and reference of original method:	Orr, P. T., and G. J. Jones. 1998. Relationship between microcystin production and cell division rates in nitrogen‐limited *Microcystis aeruginosa* cultures. Limnol. Oceanogr. 43: 1604–1614. doi: 10.4319/lo.1998.43.7.1604
Resource availability:	NA

## Method details

### Background

*Microcystis* is a genus of freshwater cyanobacteria that is frequently found in meso- to eutrophic waters. Their phylum originated some 3 billion years ago [1,2], and their photosynthetic activity prompted the oxidation of the Earth’s atmosphere [3]. Throughout time they have successfully adapted to extreme environments through processes that have allowed them to flourish in the face of increasing human impact on aquatic ecosystems [4]. Today, *Microcystis* spp. are cosmopolitan and can pose risks to human health when freshwaters affected by blooms are used for drinking, irrigation, fishing, and recreational purposes [1,5]. The distribution, frequency and intensity of blooms have increased in response to climate change factors such as elevated temperatures, CO_2_ levels, and eutrophication [6,7] which have increased risks to humans and aquatic biota. In a global analysis conducted by Harke et al. [8] on the present state of knowledge of *Microcystis* (e.g. geographic distribution, toxins, genomics, phylogeny, and ecology), blooms were reported in 108 countries, of which 79 confirmed the presence of the potent hepatotoxin microcystin.

Microcystins are cyclic heptapeptides capable of causing death to humans [9], animals [10], and aquatic invertebrates [11,12]. Upon ingestion by humans and animals, microcystins are transported to the liver where they cause toxicity by inhibiting protein phosphatases 1 and 2A [13]. The inhibition of these protein phosphatases can cause cytoskeletal degradation and breakdown of hepatocytes, causing blood to pool in the liver that is followed by hemorrhaging and organ failure [14]. Over 250 different microcystin congeners have been identified [15] whose toxicity is affected by the variation in molecular structure [16,17] and congener concentration within cyanobacterial cells is affected by changes in environmental factors including light intensity [[18], [19], [20]], pH [18], temperature [18,20], and nutrient concentrations [16,20,21]. Frequently detected and thoroughly studied among these congeners is microcystin-LR (CAS: 101043-37-2, C_49_H_74_N_10_O_12_), which is regarded as one of the most toxic forms of microcystins [8]. In fact, the widespread occurrence of microcystin-LR led the World Health Organization to institute a guideline value of 1 μg L^−1^ in drinking water [22], which is in effect in most countries.

Increasing concern over impacts from cyanobacterial harmful algal blooms has highlighted the need for studies that aim to better understand the production and dynamics of algal toxins. Culture studies continue to lay the groundwork for characterizing the relationship between *Microcystis* cell growth and microcystin toxin production over short periods of time (typically between 10–30 days) [16,19,[23], [24], [25]]. To support these studies, new culture techniques that can yield and sustain high concentrations of microcystins from *Microcystis aeruginosa* have not been explored and are of great interest. Currently, the costs to purchase microcystin analytical standards at >95 % purity can be prohibitive and may hinder researchers from designing large-scale and long-term exposure experiments. Moreover, recent work by Janssen [26] suggests that in addition to microcystins other bioactive metabolites (e.g. cyanopeptolins, aerucyclamides, aeruginosines, microginins) can also contribute to the overall toxicity of toxigenic strains of cyanobacteria found in the environment. Validating a new culture method for *M. aeruginosa* that retains microcystins and other bioactive metabolites, and demonstrating its advantage over existing techniques can, therefore, serve as a stepping-stone for researchers who would like to perform mechanistic studies, which ultimately will better define exposure-response relationships and improve our understanding of the risks posed to aquatic biota exposed to microcystins.

Here we describe a simple culture technique that circumvents the costs to purchase analytical standards and supports the production of high concentrations of microcystin-LR and its demethylated counterpart [D-Asp^3^]-microcystin-LR produced by *M. aeruginosa*. To validate our method we: 1) measured cell concentrations using traditional approaches; 2) measured the concentrations of microcystins using a high throughput method based on liquid chromatography coupled to high resolution mass spectrometry that is rapid, precise, has low detection limits and can quantify each microcystin congener; 3) measured the concentrations of cell pigments (i.e. phycobiliproteins and chlorophyll *a*) using a spectrophotometric method in order to better understand any physiological changes occurring during growth of *M. aeruginosa*; and 4) calculated the rates and maximum concentration of microcystin congener production per *M. aeruginosa* cell (referred to as cell quotas). The significance of this method is that we were able to: 1) yield microcystins at concentrations that are environmentally relevant to freshwaters around the world (e.g. 1–300 μg L^−1^); 2) maintain these concentrations without resupplying fresh medium (further reducing costs); and 3) utilize rate equations to model the relationship between the environmental conditions in the cultures and changes occurring within the *M. aeruginosa* cells.

### Origin of *Microcystis aeruginosa*

**Fig. 1:**
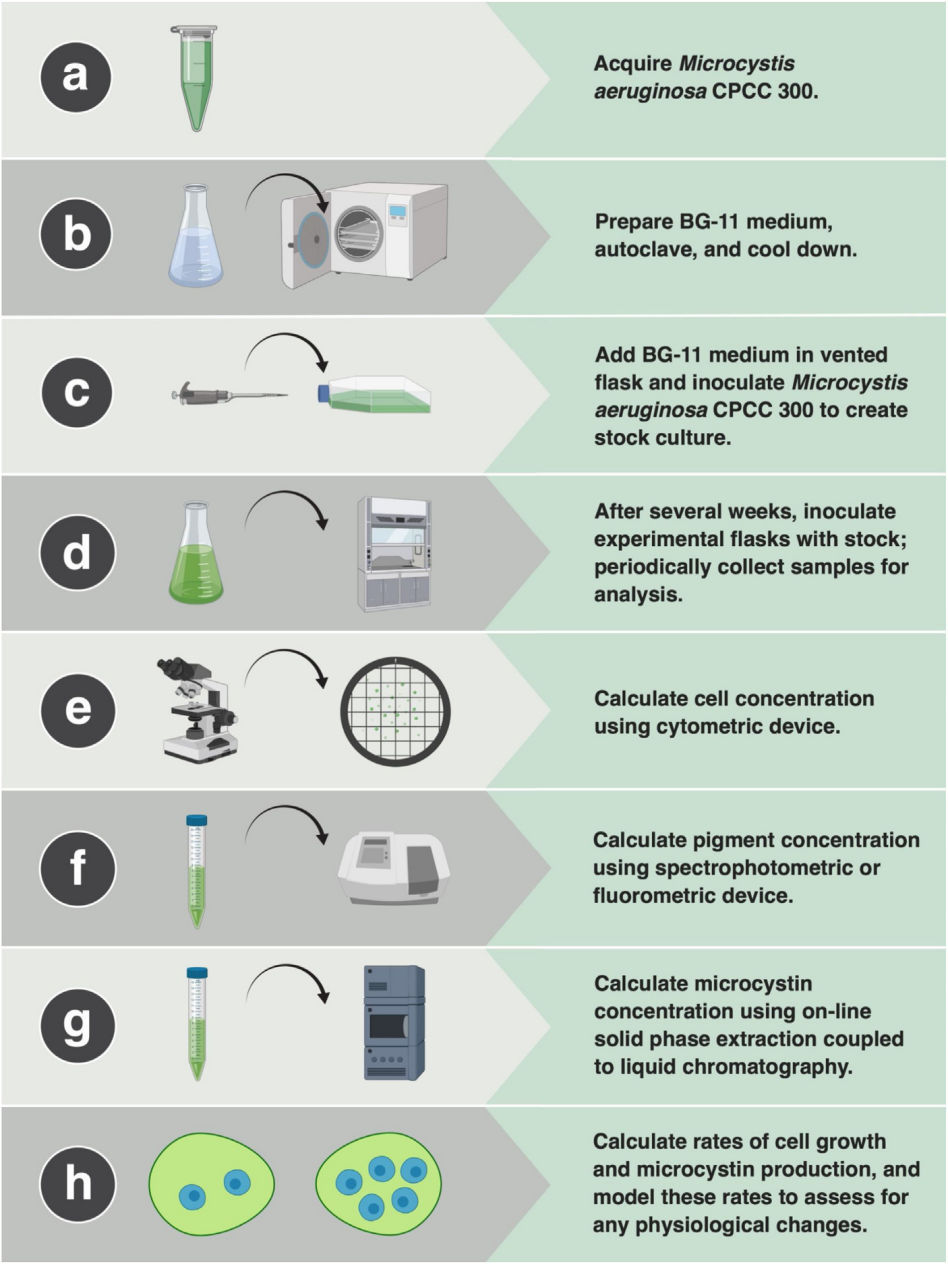
Protocol for culturing *Microcystis aeruginosa* CPCC 300.

*M. aeruginosa* strain CPCC 300 was provided by the Canadian Phycological Culture Centre (University of Waterloo, Waterloo, Canada). *M. aeruginosa* CPCC 300 originates from an extract taken from Pretzlaff Pond, Alberta, Canada, on August 7, 1990 (deposited by E. Prepas/A. Lam as #45-4A in March, 1993). *M. aeruginosa* CPCC 300 is a toxic strain that develops unicellular cells and produces two microcystin congeners, microcystin-LR and [D-Asp^3^]-microcystin-LR (Figs. 1, Fig. S1).

### Culturing of *Microcystis aeruginosa* CPCC 300

*M. aeruginosa* CPCC 300 was grown in a modified recipe of the Blue-Green-11 (BG-11) medium [27]. To prepare this medium, the BG-11 Trace Metals Solution (Table S1) was prepared by adding each of the components to approximately 900 mL of dH_2_O while continuously stirring, bringing the total volume to 1 L. Next, the BG-11 Vitamin B_12_ + Biotin Solution (Table S2) was prepared by adding 0.05 mL of Vitamin B12 and 0.5 mL of Biotin to approximately 50 mL of dH_2_O [28,29]. Thiamine hydrochloride was not added to this solution. Lastly, the BG-11 liquid media (Table S3) was prepared by adding each of the components to approximately 900 mL of dH_2_O while continuously stirring, bringing the total volume to 1 L. The pH was then adjusted to 7.5 by adding drops from 10 mM of HCl as necessary. This procedure was repeated until a sufficient volume of BG-11 medium was prepared for the method validation, upon which contents were transferred to flasks and sterilized in an autoclave for 60 min at 121 °C. After autoclaving, flasks were brought back to room temperature for a cool down period, after which 350 mL of BG-11 medium was inoculated with an initial cell concentration of 1.5 × 10^5^ cells mL^−1^ (equal to 5 mL) of *M. aeruginosa* CPCC 300 in a Falcon^TM^ 500 mL polystyrene tissue culture treated flask with a vented cap. These stock cultures were maintained for 14 d at 21.0 ± 1 °C under a 16:8 h of light:dark cycle using fluorescent bulbs (Life-GLO T-8 40 W) at 6 ± 2 μmol photons m^–2^ s^–1^. Fluorescent bulbs were 75 cm above the flasks (Fig. 1).

### Experimental design

The experimental cultures were monitored for 46 d. Five 2000 mL PYREX® Erlenmeyer flasks each received approximately 800 mL of BG-11 medium. Erlenmeyer flasks are traditionally used for culturing *M. aeruginosa* and were therefore chosen for the method validation [20,30]. Each flask was inoculated with approximately 40 mL of *M. aeruginosa* CPCC 300 from the stock cultures, the lid was tightly covered with aluminum foil, and maintained under the same environmental conditions as outlined previously for the stock cultures. Approximately midpoint during the experiment (Day 21), lids for all flasks were loosened to allow for greater atmospheric exchange to prevent cultures from being stressed. Samples were collected until there remained approximately 50 % of the original volume in each flask. On each sample collection day, and from each flask, samples were withdrawn 2 h after the light phase had commenced to estimate cell, microcystins and cellular pigment concentrations. Samples were collected under a Microzone Inc. CLASS II Type A2 Biological Safety Cabinet (Ottawa, ON, Canada) to ensure sterilization was maintained while *M. aeruginosa* CPCC 300 was being handled. To ensure homogenization prior to sample collection, flasks were swirled for 10 s. Flasks were rearranged randomly daily to reduce effects caused by minor differences in photon irradiance (Fig. 1).

### Measurement of cell concentrations by hemocytometer

To obtain an accurate estimate of cell concentrations, two 10 μL aliquots were collected and cell counts performed within an hour. Cell concentrations were determined by loading 10 μL on each grid of a Hausser Scientific Bright-Line^TM^ Hemocytometer (Horsham, PA, United States) with Propper® rectangular hemocytometer cover glass (Long Island City, NY, United States) and observing it using an Olympus® B071 BH-2 Series System Microscope (Richmond Hill, ON, Canada) at 10× magnification. The number of cells was recorded for each grid, and cell concentrations were calculated accordingly. When necessary, a dilution factor was applied. No intact dead cells were observed throughout the duration of the experiment (Fig. 1).

### Measurement of microcystins

To obtain measurements of microcystin concentrations, 5 mL was withdrawn from each flask at each time point and stored in 15 mL Corning® polypropylene centrifuge tubes at −80 °C for later analysis. Microcystins were analyzed using on-line solid phase extraction coupled to liquid chromatography-quadrupole time-of-flight high resolution mass spectrometry (Waters Xevo G2-XS, Milford, MA, USA), optimized by Ortiz et al. [31]. In this targeted and non-targeted high throughput method, samples of *M. aeruginosa* CPCC 300 at each time point were measured and 12 microcystin variants (LR, YR, RR, HtyR, HilR, WR, LW, LA, LF, LY, Dha_7_-LR, and Dha_7_-RR) and anatoxin-A (all standards purchased from Enzo Life Sciences, Farmingdale, NY, USA) were quantified using nodularin as the internal standard. Using this high throughput method, batches of 50 samples can be prepared for instrumental analysis in less than 3 h. Detection limits were 0.05 μg L^−1^ with an expanded uncertainty ranging from 4 to 14 % for the different variants, which takes into account the uncertainty coming from the sample preparation, the instrument, and calibration standards. The microcystin cell quota (*Q*_mcyst_), expressed as femtograms (fg) microcystin per cell, was calculated by dividing the concentration of each detectable microcystin congener (fg mL^−1^) by the cell concentration (cells mL^−1^) (Fig. 1).

### Measurement of phycobiliproteins and chlorophyll *a*

For cellular pigment determinations, 5 mL was withdrawn for analysis of phycobiliproteins and chlorophyll *a* by ultraviolet-visible spectrophotometry. Phycobiliprotein and chlorophyll *a* content was quantified using a Molecular Devices LLC SPECTRAmax PLUS 384 Microplate Reader (San Jose, CA, United States) with SoftMax® Pro 6.3, Microplate Data Acquisition and Analysis Software (Sunnyvale, CA, United States). Optical density (OD) between 200–800 nm was captured, and the following absorbance maxima were recorded for each class of phycobiliproteins: allophycocyanin (λ_max_ = 650 nm, purple); phycocyanin (λ_max_ = 610–625 nm, blue); and, phycoerythrin (λ_max_ = 550–565 nm, red). The OD for chlorophyll *a* was measured at λ_max_ = 665 nm. Phycobiliprotein and chlorophyll *a* concentrations were calculated at 650 nm for allophycocyanins (AP), 620 nm for phycocyanins (PC), 565 nm for phycoerythrins (PE); and, 665 nm for chlorophyll *a* (chl *a*) using associated Eqs. (1), (2), and (3) described by Tandeau de Marsac and Houmard [30] and (4) described by Tailing and Driver [32] (Fig. 1).

(1) $$\text{A}\text{P}\;(\text{m}\text{g}\;{\text{m}\text{L}}^{-1})=\;\frac{{\text{O}\text{D}}_{650\text{n}\text{m}}-0.19\;\times \;{\text{O}\text{D}}_{620\text{n}\text{m}}}{5.65}$$

(2) $$\text{P}\text{C}\;(\text{m}\text{g}\;{\text{m}\text{L}}^{-1})=\;\frac{{\text{O}\text{D}}_{620\text{n}\text{m}}-0.7\;\times \;{\text{O}\text{D}}_{650\text{n}\text{m}}}{7.38}$$

(3) $$\text{P}\text{E}\;(\text{m}\text{g}\;{\text{m}\text{L}}^{-1})=\;\frac{{\text{O}\text{D}}_{565\text{n}\text{m}}-2.8\;\left[\text{P}\text{C}\right]-\;1.34\;[\text{A}\text{P}]}{12.7}$$

(4) $$\text{c}\text{h}\text{l}\;a\;(\text{μ}\text{g}\;{\text{m}\text{L}}^{-1})=\;{\text{O}\text{D}}_{665\text{n}\text{m}}\;\times \;13.9$$

The pigment cell quota (*Q*_pigment_) —expressed as picogram (pg) pigment per cell— was calculated by dividing the concentration of each pigment (pg mL^−1^) by the cell concentration (cells mL^−1^).

### Calculation of rates

First order rate kinetics provided by Orr et al. [25] were used to model the relationship between the environmental conditions in the cultures and the physiological changes in *M. aeruginosa* cells and production of microcystins (Fig. 1).

Using the first order rate constant (*μ*), the initial concentration (*C*_0_), the final concentration (*C*_1_), and time intervals (*t*_0_ and *t*_1_), the rate of cell growth (*μ*_growth_) and rate of microcystin congener production (*μ*_mcyst_) were calculated using Eq. (5).

(5) $$\mu =\;\frac{\text{ln}\left(C_{1}\right)-\text{ln}\left(C_{0}\right)}{t_{1}-t_{0}}$$

The relationship between the rate of cell growth (*μ*_growth_) and rate of microcystin congener production (*μ*_mcyst_) were further investigated using Eqs. (6), (7), and (8).

(6) $$0.5\;<\;\frac{\mu_{\text{m}\text{c}\text{y}\text{s}\text{t}}}{\mu_{\text{g}\text{r}\text{o}\text{w}\text{t}\text{h}}}<1$$

Eq. (6) declares the rate of microcystin congener production (*μ*_mcyst_) between *t*_0_ and *t*_1_ is slower than the rate of cell growth (*μ*_growth_), where 0.5 means no production is occurring and the existing microcystins are being diluted as cells continue to grow (i.e. cells are becoming less toxic).

(7) $$\frac{\mu_{\text{m}\text{c}\text{y}\text{s}\text{t}}}{\mu_{\text{g}\text{r}\text{o}\text{w}\text{t}\text{h}}}=1$$

Eq. (7) declares the rate of microcystin congener production (*μ*_mcyst_) between *t*_0_ and *t*_1_ is equal to the rate of cell growth (*μ*_growth_).

(8) $$\frac{\mu_{\text{m}\text{c}\text{y}\text{s}\text{t}}}{\mu_{\text{g}\text{r}\text{o}\text{w}\text{t}\text{h}}}>1$$

Eq. (8) declares the rate of microcystin congener production (*μ*_mcyst_) between *t*_0_ and *t*_1_ is faster than the rate of cell growth (*μ*_growth_) (i.e. cells are becoming more toxic).

### Measurement of nutrients and metals

Samples of BG-11 medium were submitted to Ontario’s Ministry of the Environment, Conservation and Parks (MECP) Laboratory Services Branch (Etobicoke, ON, Canada) for nutrient and metals analysis (Table S4).

### Data analysis

To characterize changes in the concentration of cells, toxins and pigments, linear and nonlinear models were applied to all data (i.e. *t*_0_ to *t*_46_) and the Akaike Information Criterion (AICc) was used to determine the best fit. Based on the lowest AICc, a nonlinear regression exponential growth, single, 3 parameter equation (*f* = y_0_ + a*e^bx^*; α = 0.05) was chosen to describe changes in cell concentration (cells mL^−1^), microcystin congener concentration (μg L^−1^), pigment concentration (μg mL^−1^), microcystin cell quota (*Q*_mcyst_, fg cell^−1^), and pigment cell quota (*Q*_pigment_, pg cell^−1^) over time. Correlations between rates (*μ*) and cell quotas (*Q*) were also calculated using Pearson’s product moment correlation coefficient (α = 0.05). Data were divided into three groups (*t*_0_ to *t*_11_, *t*_12_ to *t*_21_, and *t*_25_ to *t*_46_) demarcated by distinct increases or decreases in the relative rates of cell growth (*μ*_growth_) and microcystin congener production (*μ*_mcyst_) between *t*_0_ to *t*_46_ to account for possible changes in cell physiology that may have occurred over the course of the method validation. Data was tested for normality using the Shapiro-Wilk’s test (α = 0.05). When normality failed (*p* < 0.05), Kruskal-Wallis One Way Analysis of Variance (ANOVA) on Ranks (α = 0.05) was conducted between the three populations for the rates of cell growth (*μ*_growth_), microcystin congener production (*μ*_mcyst_), and pigment production (*μ*_pigment_). Linear regression was also performed to measure the extent that there was a linear relationship between the independent variable (*μ*_growth_) and dependent variables (*μ*_mcyst_, *μ*_pigment_). When the Shapiro-Wilk’s test failed (*p* < 0.05), a post hoc non-parametric pairwise multiple comparisons procedure was conducted using Dunn’s method. Statistical analyses were performed using Sigma Stat (Version 4.0, Systat Software, San Jose, CA, US).

### Method validation

**Fig. 2:**
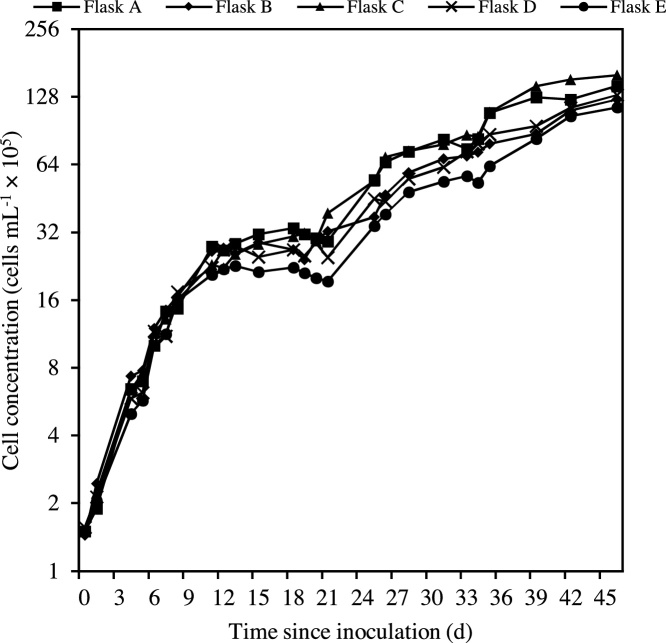
Logarithmic growth curves for the five experimental cultures of *Microcystis aeruginosa* CPCC 300 since time of inoculation.

Cell concentrations (cells mL^−1^) increased in all cultures from *t*_0_ to *t*_11_, with growth rates slowly decreasing to near zero by *t*_11_. From *t*_12_ to *t*_21_ the average growth rate was 0.02 cells d^−1^ and there was a gradual decline in cell concentrations in all cultures. From *t*_25_ to *t*_46_, growth rates increased, ranging between 0.03 and 0.17 cells d^−1^. By *t*_46_ the cultures had a fresh blue-green colour and maintained this colour for 6 months. After *t*_11_, some divergence between flasks was observed but the maximum difference between the greatest and smallest cell counts was <2 % (Fig. 2). Although changes in which flasks contained the greater number of cells differed between *t*_11_ and *t*_46_, this had little effect on microcystin concentrations, which was significantly and positively correlated with cell concentrations ( *r*^2^ = 0.970, p < 0.005). Collectively, this indicates consistent performance (i.e. growth and toxin production) of the cultures over the entire duration of the experiment.

**Fig. 3:**
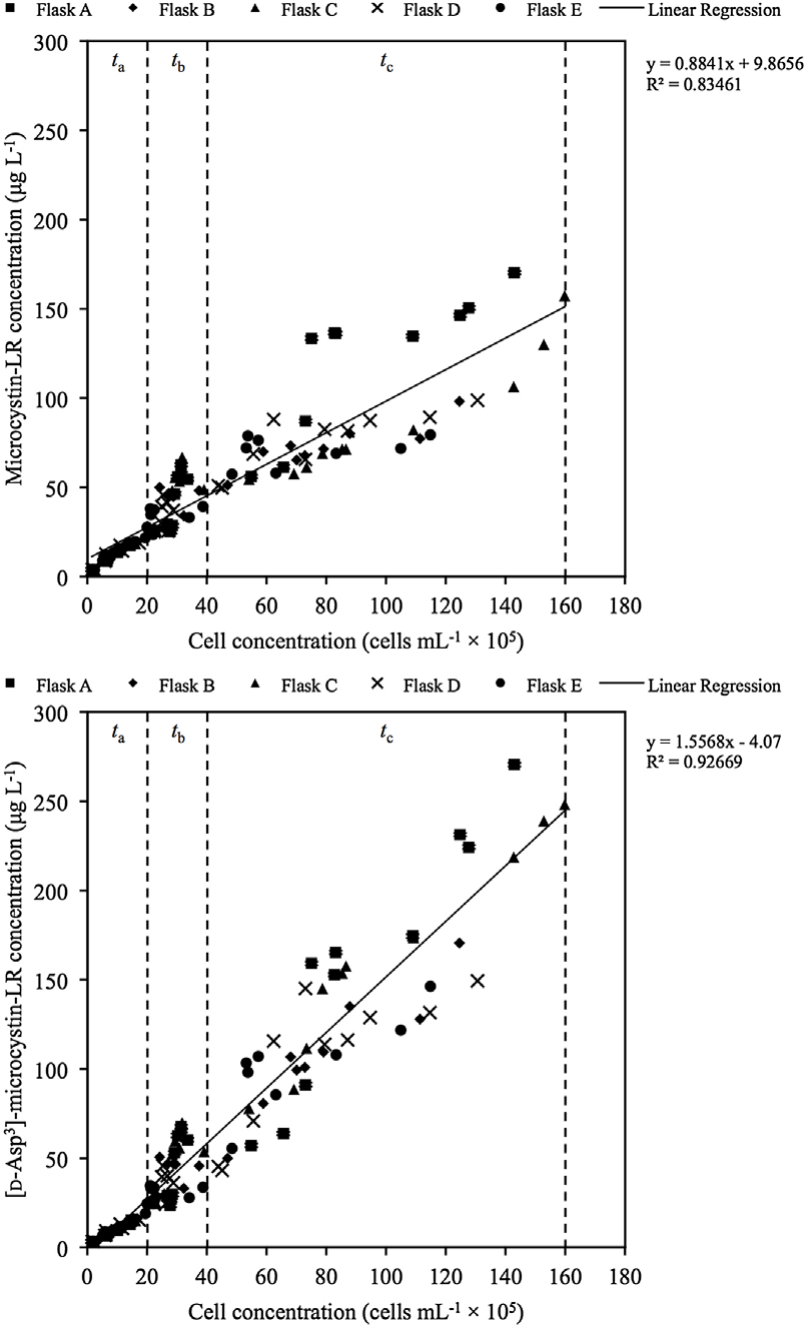
Detectable microcystin-LR (top) and [D-Asp^3^]-microcystin-LR (bottom) over cell concentrations in five experimental cultures of *Microcystis aeruginosa* CPCC 300 since time of inoculation. The microcystin cell quota (*Q*_mcyst_) increases above and decreases below the linear regression line. *t*_a_ = days 0–11; *t*_b_ = days 12–21; *t*_c_ = days 25–46.

**Fig. 4:**
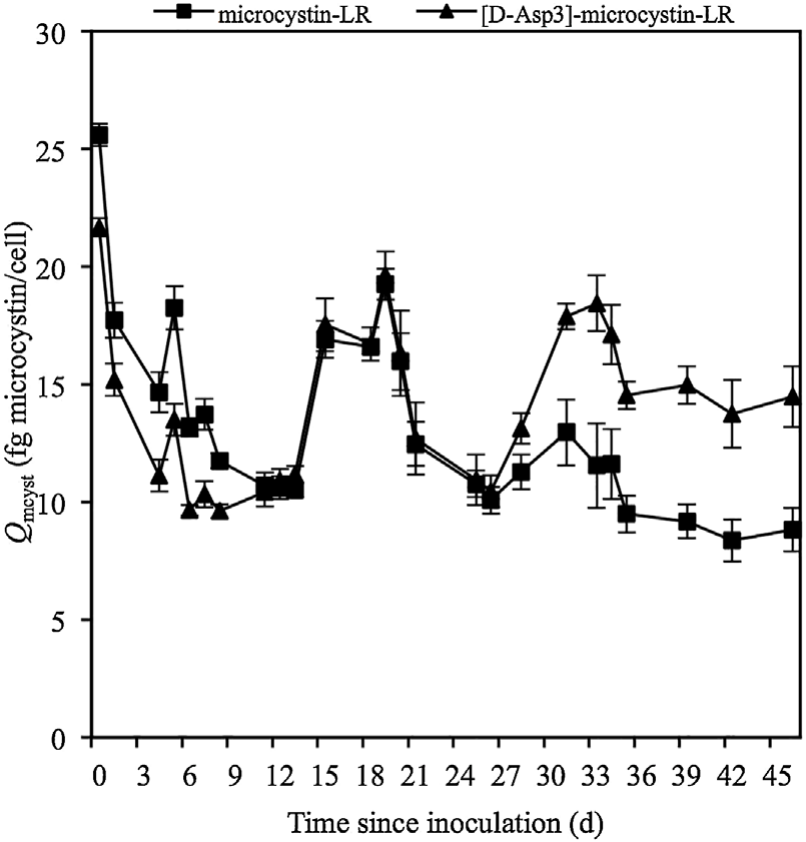
Microcystin cell quotas (*Q*_mcyst_) averaged for microcystin-LR and [D-Asp^3^]-microcystin-LR, respectively, produced by *Microcystis aeruginosa* CPCC 300 since time of inoculation.

Microcystin concentrations (μg L^−1^) also increased in all cultures from *t*_0_ to *t*_11_; however, unlike cell concentrations there was a briefer plateau (*t*_12_ to *t*_14_) followed by a short increase (*t*_15_ to *t*_19_) and then decrease (*t*_20_ to *t*_21_) in microcystin content (Fig. 3). Notwithstanding, the cultures experienced a recovery and increased after *t*_21_. Chemical analyses also confirmed that microcystins were intracellular throughout the duration of the experiment, and cell counts by hemocytometer did not detect dead cells, suggesting that despite the brief plateau observed between *t*_12_ to *t*_19_ *M. aeruginosa* cells were viable and intact. As for the predominance of microcystin congeners: from *t*_0_ to *t*_11_ the concentration of microcystin-LR was higher than [D-Asp^3^]-microcystin-LR; from *t*_12_ to *t*_21_ both congeners were approximately equal; and, from *t*_25_ to *t*_46_ [D-Asp^3^]-microcystin-LR exceeded microcystin-LR. Microcystin cell quotas (*Q*_mcyst_) also exhibited comparable trends in the shift of predominance in microcystin congeners: from *t*_0_ to *t*_11_ the average ratio of *Q*_mcyst-LR_:*Q*_mcyst-dmLR_ was 16:13 fg microcystin cell^−1^; from *t*_12_ to *t*_21_ the average ratio of *Q*_mcyst-LR_:*Q*_mcyst-dmLR_ was 15:15 fg microcystin cell^−1^; and, from *t*_25_ to *t*_46_ the average ratio of *Q*_mcyst-LR_:*Q*_mcyst-dmLR_ was 10:15 fg microcystin cell^−1^ (Fig. 4). Further, a significant negative correlation between microcystin-LR cell quotas (*Q*_mcyst-LR_) and cell concentrations was found from *t*_0_ to *t*_11_ ( *r*^2^ = 0.663, *p* = 0.0139), no significant correlation was found from *t*_12_ to *t*_21_ ( *r*^2^ = 0.116, *p* = 0.455), and a significant negative correlation was found from *t*_25_ to *t*_46_ ( *r*^2^ = 0.498, *p* = 0.0225). For correlations between [D-Asp^3^]-microcystin-LR cell quotas (*Q*_mcyst-dmLR_) and cell concentrations, a significant negative correlation was found from *t*_0_ to *t*_11_ ( *r*^2^ = 0.490, *p* = 0.0535) but not from *t*_12_ to *t*_21_ ( *r*^2^ = 0.100, *p* = 0.489) or *t*_25_ to *t*_46_ ( *r*^2^ = 0.047, *p* = 0.549). This shows that while in the first half of the experiment *Q*_mcyst-LR_ and *Q*_mcyst-dmLR_ decreased as cell concentrations increased (i.e. cell growth outpaced toxin production), in the second half of the experiment *Q*_mcyst-LR_ continued to exhibit a negative correlation with cell concentrations while *Q*_mcyst-dmLR_ had no significant correlation with cell concentrations. The mean pigment cell quota (*Q*_pigment_) experienced similar trends to the mean microcystin cell quota (*Q*_mcyst_) throughout the experiment (data not shown). Among all pigments, chlorophyll *a* cell quota (*Q*_chlorophylla_) showed a significant linear correlation with *Q*_mcyst-LR_ ( *r*^2^ = 0.350, *p* < 0.001) and *Q*_mcyst-dmLR_ ( *r*^2^ = 0.240, *p* = 0.003) from *t*_0_ to *t*_46_.

Correlation of the rates of cell growth (*μ*_growth_), microcystins (*μ*_mcyst_) and cell quotas (*Q*_mcyst_) of the cultures were determined for the three time periods: from *t*_0_ to *t*_11_, *t*_12_ to *t*_21_, and *t*_25_ to *t*_46_, respectively (Table 1). From *t*_0_ to *t*_11_, there is no correlation between *μ*_growth_ and *Q*_mcyst-LR_ ( *r*^2^ = 0.001, *p* =  0.942) and *Q*_mcyst-dmLR_ ( *r*^2^ = 0.002, *p* = 0.926). However, from *t*_12_ to *t*_21_ there is a significant negative linear correlation between *μ*_growth_ and *Q*_mcyst-LR_ ( *r*^2^ = 0.676, *p* = 0.023) and *Q*_mcyst-dmLR_ ( *r*^2^ = 0.676, *p* = 0.023). As the cultures progressed into *t*_25_ to *t*_46_, *Q*_mcyst-LR_ resumed a very weak correlation with *μ*_growth_ ( *r*^2^ = 0.032, *p* = 0.622) while *Q*_mcyst-dmLR_ maintained a significant negative linear correlation *μ*_growth_ ( *r*^2^ = 0.446, *p* = 0.035). The correlation between *Q*_mcyst-LR_ and *Q*_mcyst-dmLR_ also varies at each time point: from *t*_0_ to *t*_11_ there was a significant linear correlation ( *r*^2^ = 0.929, *p* < 0.001); from *t*_12_ to *t*_21_ there was another significant linear correlation ( *r*^2^ = 0.996, *p* < 0.001); and, from *t*_25_ to *t*_46_ there was a weak correlation ( *r*^2^ = 0.204, *p* = 0.189). This indicates two findings: 1) as *μ*_growth_ decreased from *t*_12_ to *t*_21_ *Q*_mcyst_ increased; and 2) the relationship between *Q*_mcyst-LR_ and *Q*_mcyst-dmLR_ becomes uncorrelated after *t*_21_ when the lid for each culture flask was loosened to allow for greater atmospheric exchange.

**Table 1:** Correlation analyses between rates of cell growth (*μ*_g_) and microcystins (*μ*_mcyst_) and cell quotas (*Q*_mcyst_) of the experimental cultures tested by Pearson’s product moment correlation coefficient.

	Slope of regression line (*B* ± SE)	Correlation coefficient (*r*)	Coefficient of determination (*r*^2^)	Strength	Significance
*μ*_growth_ vs *μ*_mcyst-LR_					
*t_0-11_*	−0.204 ± 0.125	−0.245	0.060	Very weak	*p* = 0.597
*t_12-21_*	−1.767 ± 0.134	−0.366	0.134	Weak	*p* = 0.420
*t_25-46_*	0.154 ± 0.054	0.162	0.026	Very weak	*p* = 0.654
*μ*_growth_ vs *μ*_mcyst-dmLR_					
*t_0-11_*	−0.305 ± 0.092	−0.459	0.211	Weak	*p* = 0.300
*t_12-21_*	−1.569 ± 0.168	−0.315	0.099	Weak	*p* = 0.492
*t_25-46_*	0.298 ± 0.074	0.227	0.052	Very weak	*p* = 0.529
*μ*_growth_ vs Δ *Q*_mcyst-LR_					
*t_0-11_*	0.684 ± 3.110	0.034	0.001	None	*p* = 0.942
*t_12-21_*	−86.593 ± 2.130	−0.822	0.676	Strong	*p* = 0.023
*t_25-46_*	−4.764 ± 1.529	−0.178	0.032	Very weak	*p* = 0.622
*μ*_growth_ vs Δ *Q*_mcyst-dmLR_					
*t_0-11_*	−0.655 ± 2.327	−0.044	0.002	None	*p* = 0.926
*t_12-21_*	−86.063 ± 2.115	−0.822	0.676	Strong	*p* = 0.023
*t_25-46_*	−32.831 ± 2.131	−0.668	0.446	Moderate	*p* = 0.035
*μ*_mcyst-LR_ vs Δ *Q*_mcyst-LR_					
*t_0-11_*	5.953 ± 3.016	0.247	0.061	Very weak	*p* = 0.593
*t_12-21_*	6.693 ± 3.560	0.307	0.094	Weak	*p* = 0.503
*t_25-46_*	8.409 ± 1.483	0.299	0.089	Very weak	*p* = 0.402
*μ*_mcyst-LR_ vs Δ *Q*_mcyst-dmLR_					
*t_0-11_*	−2.189 ± 2.312	−0.121	0.015	Very weak	*p* = 0.795
*t_12-21_*	7.381 ± 3.495	0.341	0.116	Weak	*p* = 0.455
*t_25-46_*	−24.936 ± 2.509	−0.481	0.231	Weak	*p* = 0.159
*μ*_mcyst-dmLR_ vs Δ *Q*_mcyst-LR_					
*t_0-11_*	−0.0973 ± 3.112	0.003	0.000	None	*p* = 0.995
*t_12-21_*	4.060 ± 3.671	0.192	0.037	Very weak	*p* = 0.680
*t_25-46_*	7.242 ± 1.452	0.356	0.127	Weak	*p* = 0.313
*μ*_mcyst-dmLR_ vs Δ *Q*_mcyst-dmLR_					
*t_0-11_*	−7.509 ± 2.197	−0.333	0.111	Weak	*p* = 0.466
*t_12-21_*	4.739 ± 3.621	0.226	0.051	Very weak	*p* = 0.626
*t_25-46_*	−11.900 ± 2.714	−0.318	0.101	Weak	*p* = 0.371
Δ *Q*_mcyst-LR_ vs Δ *Q*_mcyst-dmLR_					
*t_0-11_*	1.118 ± 1.379	0.964	0.929	Strong	*p* < 0.001
*t_12-21_*	1.005 ± 0.222	0.998	0.996	Strong	*p* < 0.001
*t_25-46_*	0.246 ± 1.386	0.452	0.204	Weak	*p* = 0.189

None = *r* = 0.00; Very weak = *r* < 0.30; Weak = 0.30 < *r* 0.50; Moderate = 0.50 < *r* < 0.70; Strong = *r* > 0.70.

Linear regression and standard errors were calculated by the least squares method, df = 124.

Application of first order rate kinetics to assess the relationship between the production of microcystin-LR (*μ*_mcyst-LR_) and [D-Asp^3^]-microcystin-LR (*μ*_mcyst-dmLR_) over cell growth (*μ*_growth_) was calculated from *t*_1_ to *t*_46_ (Table S5). Of interest is between *t*_12_ to *t*_19_ when *M. aeruginosa* cells were exhibiting symptoms of stress. As *μ*_mcyst-LR_ and *μ*_mcyst-dmLR_ increased at similar rates from *t*_12_ to *t*_19_, microcystin-LR cell quota (*Q*_mcyst-LR_) and [D-Asp^3^]-microcystin-LR (*Q*_mcyst-dmLR_) also increased. This suggests that during this time period *M. aeruginosa* cells were stressed and toxin production outpaced cell growth. However, after *t*_21_ —when the lid for each culture flask was loosened to allow for greater atmospheric exchange— *μ*_mcyst-LR_ and *μ*_mcyst-dmLR_ began to stabilize at rates similar to cell growth. Furthermore, as the rate of microcystin production began to stabilize with cell growth, *μ*_mcyst-dmLR_ surpassed *μ*_mcyst-LR_.

## Additional information

For over 60 years, researchers have attempted to decipher the conditions that enable the production and cessation of microcystins by freshwater cyanobacteria [23]. Numerous reports affirm the production of microcystins by *M. aeruginosa* is linked to physiological and environmental processes including cell division [25,33], light intensity and photoperiod [[18], [19], [20],^34^], temperature [18,20], ultraviolet [35], and the availability of nutrients [16,20]. To advance further understanding, the need to optimize conditions for culturing *M. aeruginosa* to produce and sustain high concentrations of microcystins, while being cost-effective, is an important challenge facing researchers. In this experiment, the culture conditions adopted and strain of *M. aeruginosa* chosen produced high concentrations of microcystin-LR (120.75 μg L^−1^) and [D-Asp^3^]-microcystin-LR (196.94 μg L^−1^) for 46 d in the laboratory. Despite popular claims that metabolic end products will deteriorate the growth of cells in laboratory cultures, our results corroborate with those of Lyck [19] who found that this does not hold true for *M. aeruginosa* strains CYA 228 and PCC 7806. The strain selected for our method validation, *M. aeruginosa* CPCC 300, has shown it requires low maintenance and does not need a resupply of fresh medium in order to consistently produce microcystins at concentrations that are relevant both to freshwaters that regularly experience *Microcystis* blooms (i.e. 1–300 μg L^−1^) and the guideline value of 1 μg L^−1^ in drinking water instituted by the World Health Organization. In others experiments conducted by our laboratory, *M. aeruginosa* CPCC 300 has also shown the potential to: 1) produce microcystin-LR and [D-Asp^3^]-microcystin-LR concentrations totaling as high as 1200 μg L^−1^; 2) maintain these concentrations for up to 6 months in stationary phase without resupply of fresh medium; and 3) remain stable for over 18 months when frozen at −80 °C (R.S. Shahmohamadloo, University of Guelph, Guelph, ON, Canada, unpublished data). This information will be important for researchers who would like to stockpile fresh or frozen microcystins for future large-scale and long-term experiments.

While *M. aeruginosa* CPCC 300 has relatively high microcystin cell content with stable proportions of microcystin-LR and [D-Asp^3^]-microcystin-LR [34], our findings indicate a decline from *t*_12_ to *t*_21_ and a shift in the proportion of congeners from *t*_25_ to *t*_46_. Certain parameters can be ruled out to guide our postulation for this decline and shift in microcystins: light intensity, photoperiod, and temperature. These parameters were constant throughout the method validation and correspond with freshwaters conditions during a typical harmful algal bloom season [34]. Light limitation due to self-shading of *M. aeruginosa* cells was also considered, however given the cell concentrations increased markedly after *t*_21_ it is unlikely this parameter caused a decrease in the concentration of microcystins. Another factor that can be ruled out is lack of ultraviolet light because the fluorescent bulbs were selected that include UV light and mimic the wavelength range that is needed for *M. aeruginosa* cell pigments (phycobiliproteins and chlorophyll *a*) to grow. Further, it is unlikely that nutrient limitation was a factor; if this were the case, we would have seen a continued decline in cell, microcystin, and pigment concentrations after *t*_21_. It is important to note that the experimental cultures were not resupplied with fresh medium, and subsamples were regularly taken out, thus causing a decrease in nutrients (e.g. nitrogen, phosphorus) over the course of the experiment. If nutrient limitation were a cause for the decline in microcystin concentrations seen from *t*_12_ to *t*_21_, it should have continued to *t*_46_. Instead, the experimental cultures responded favourably to the perturbation on *t*_21_ when atmospheric carbon exchange increased, and recovered, albeit not at the same rate of microcystin production (*μ*_mcyst_) but along similar patterns of growth. This points to the role of dissolved inorganic carbon (e.g. carbon dioxide, CO_2_; carbonic acid, H_2_CO_3_; bicarbonate, HCO_3_^−^; and carbonate, CO_3_^2-^) as the parameter that likely caused the decline of microcystins from *t*_12_ to *t*_21_.

Two studies have previously investigated the effects of inorganic carbon concentrations on the relative abundance of microcystin congeners [36,37]. Sandrini et al. [36] investigated the response of *M. aeruginosa* strain PCC 7806 to rising levels of CO_2_, and how the production of microcystins and cell physiology is altered in the presence of elevated CO_2_ partial pressure (pCO_2_). Many cyanobacteria including *Microcystis* have evolved highly advanced CO_2_-concentrating mechanisms that include CO_2_ and bicarbonate uptake systems [3,36]. These mechanisms are important for *Microcystis* to grow and spread rapidly in supersaturated lakes that contain high levels of dissolved CO_2_, sometimes exceeding equilibrium with the atmosphere [38]. To test this hypothesis, Sobek et al. [38] cultured *M. aeruginosa* strain PCC 7806 in controlled laboratory chemostats that were changed from low pCO_2_ (200 ppm) to high pCO_2_ (1450 ppm) conditions that are representative of supersaturated lakes. Results confirmed that *M. aeruginosa* cells benefited strongly from increased dissolved CO_2_. Elevated CO_2_ levels alleviated *M. aeruginosa* cells from inorganic carbon limitation, which led to a higher population abundance, increased buoyancy in the cells, and about a 2.5-fold increase in microcystin cell quota (*Q*_mcyst_). Another study used a similar set-up but instead provided nitrogen-limited conditions and exposed three strains of *M. aeruginosa* to low pCO_2_ (400 ppm) and high pCO_2_ (1200 ppm); Liu et al. [37] hypothesized that elevated pCO_2_ would increase the cellular C:N ratios in *M. aeruginosa*, and that a higher C:N ratio would promote the production of the more toxic microcystin congeners. Results confirmed that microcystin congeners with higher C:N ratios (e.g. microcystin-LW, microcystin-LF) increased with elevated pCO_2_, and vice versa (e.g. microcystin-RR, microcystin-LR). Liu et al. [37] suggest that *Microcystis* may preferentially produce more toxic microcystin congeners in elevated pCO_2_ under nitrogen-limited conditions. Our results corroborate these findings. In fact, when viewed from *t*_0_ to *t*_46_, the microcystin cell quotas for microcystin-LR (*Q*_mcyst-LR_) and [D-Asp^3^]-microcystin-LR (*Q*_mcyst-dmLR_) were highest between *t*_12_ to *t*_21_ when the C:N ratio was assumed to be lower than between *t*_0_ and *t*_11_. This further confirms that nitrogen was not a limiting nutrient in our experiment and did not inhibit the production of microcystins.

Although it is well documented in the literature that CO_2_ can influence the production of microcystins by *M. aeruginosa*, as far as we can ascertain no studies have evaluated the role of carbon in relation to congener production. While serendipitous, our results demonstrate that carbon limitation can influence the production of [D-Asp^3^]-microcystin-LR over microcystin-LR when other relevant environmental parameters are constant (Fig. 4). Our findings contrast with the concluding remarks of Orr et al. [25] who suggest that microcystin production is not triggered by environmental stressors. Environmental stressors are believed to drive changes in *Microcystis* bloom toxicity by affecting the rates of cell growth (*μ*_growth_) and microcystin cell quotas (*Q*_mcyst_), but do not trigger any effect on the pathway in which microcystin congeners are produced [25]. We provide evidence that under carbon-limited conditions the following can occur: as *μ*_growth_ decreases, *Q*_mcyst_ increases ( *r*^2^ = 0.676, *p* =  0.023); the relationship between *Q*_mcyst-LR_ and *Q*_mcyst-dmLR_ will be tightly correlated in carbon-stressed situations ( *r*^2^ = 0.996, *p* <  0.001), however after this stress is relieved and carbon is no longer limited, *Q*_mcyst-LR_ and *Q*_mcyst-dmLR_ will be uncorrelated (*r*^2^ = 0.204, *p* =  0.189); and, the average ratio of *Q*_mcyst-LR_:*Q*_mcyst-dmLR_ can shift in dominance depending on the extent to which atmospheric carbon exchange is limited, which in our case showed a shift from 16:13 fg microcystin cell^−1^ (*t*_0_ to *t*_11_) to 15:15 fg microcystin cell^−1^ (*t*_12_ to *t*_21_) to 10:15 fg microcystin cell^−1^ (*t*_25_ to *t*_46_). However, given the known abundance of CO_2_ in freshwaters, field data is a necessary point for comparison to assess the relevance of our discovering a shift in microcystin congener production. To substantiate our discovery, we received water samples from the Great Lakes and inland waters across Ontario, Canada, during harmful algal blooms season from 2016 to 2018. We postulated that if microcystin-LR and [D-Asp^3^]-microcystin-LR were present in samples, the ratio of microcystin-LR would be greater because atmospheric carbon exchange was abundant. Results confirmed the presence of both microcystin congeners across the samples received, and found the concentration of microcystin-LR was higher than [D-Asp^3^]-microcystin-LR after quantification by on-line solid phase extraction coupled to liquid chromatography-quadrupole time-of-flight high resolution mass spectrometry (R.S. Shahmohamadloo, University of Guelph, Guelph, ON, Canada, unpublished data). Our field results highlight the need to maintain culture conditions in order that *M. aeruginosa* can produce microcystin-LR and [D-Asp^3^]-microcystin-LR at ratios comparable to those found in the environment. To ensure uninterrupted growth of *M. aeruginosa* and high concentrations of microcystins in the laboratory, we recommend experimental cultures flasks be sufficiently ventilated. Cell culture flasks with vented caps —filled no more than 50 % of the flask volume to allow for sufficient air exchange— are an excellent and cost-effective approach to maintaining cell growth and producing microcystins at a range between 300 to 1200 μg L^−1^, as has been the case in our laboratory (R.S. Shahmohamadloo, University of Guelph, Guelph, ON, Canada, unpublished data). Furthermore, cell culture flasks with vented caps provide greater stability (i.e. little to no stress) and reduce the variability in production of microcystins among replicates when compared to Erlenmeyer flasks, as was the case in our experiment.

Failure to allow for sufficient atmospheric carbon exchange within culture flasks can increase the overall toxicity of *M. aeruginosa* CPCC 300. In one study, Shimizu et al. [39] assessed the cytotoxicities of 16 microcystin congeners on primary cultured rat hepatocytes by measuring cellular adenosine triphosphate content, followed by calculating the inhibitory concentration at 50 % (IC_50_). Results showed [D-Asp^3^]-microcystin-LR was approximately four times more cytotoxic (IC_50_ = 0.217 μg mL^−1^) than microcystin-LR (IC_50_ = 0.800 μg mL^−1^) to rat hepatocytes. Although lethal dose toxicity information is currently only available for microcystin-LR (LD_50_ = 50 μg kg^-1^ intraperitoneal injection in mice, [37]), the potential exists for [D-Asp^3^]-microcystin-LR to inflict with greater effect liver and kidney damage, tumour promotion, gastroenteritis, reduced DNA repair and reproductive toxicity in mammals and humans [3]. If this is the case, strains of *Microcystis* that are similar to *M. aeruginosa* CPCC 300 may have the ability to favour the production of [D-Asp^3^]-microcystin-LR in low pCO_2_ (<400 ppm) and microcystin-LR in high pCO_2_ (>1200 ppm) environments, respectively, which has implications for the overall toxicity of microcystins in experiments conducted in the laboratory and field.

## Conclusion

This method presents a cost-effective culture technique that can yield and sustain high concentrations of microcystin-LR (120.75 μg L^−1^) and [D-Asp^3^]-microcystin-LR (196.94 μg L^−1^) produced by *M. aeruginosa* CPCC 300 when relevant environmental conditions are controlled in the laboratory. In the face of adversity from *t*_12_ to *t*_21_, we discovered *M. aeruginosa* CPCC 300 are resilient in carbon-limited situations and may respond to stress by shifting the ratio of microcystin congeners. Future work is needed to explore the impact of nutrient modifications on the profile of microcystin congeners produced by cyanobacterial species, using both laboratory cultures and field samples. Given the variability in toxicity of microcystin congeners, a greater understanding of which and how environmental variables can regulate the abundance of microcystins will help predict the periods of greatest risk to users of freshwaters who may be exposed to these toxins through cyanobacterial harmful algal blooms [16,24,25]. We recommend the use of *M. aeruginosa* CPCC 300 as a reliable strain that can yield microcystins at concentrations that are environmentally relevant to freshwaters (e.g. 1–300 μg L^−1^). Adopting this culture technique and strain of *M. aeruginosa* can support the work of future researchers who require a continual supply of microcystin congeners to perform toxicological studies on aquatic biota at concentrations relevant to freshwater ecosystems.

## Author’s contributions

R.S.S., R.C., D.G.P. and P.K.S. conceived and designed the experiment. R.S.S. collected the data. R.S.S., X.O., C.H. analyzed the data. R.S.S. wrote the paper. All authors read, amended, and approved the final manuscript.
