# Supplementary material for: An efficient and affordable laboratory method to produce and sustain high concentrations of microcystins by Microcystis aeruginosa
Source: MethodsX. 2019 Oct 31;6:2521–35. doi: 10.1016/j.mex.2019.10.024 (PMC6861626; doi:10.1016/j.mex.2019.10.024)

**Supplementary Information**

**An efficient and affordable laboratory method to produce and sustain high concentrations of microcystins by *Microcystis aeruginosa***

René S. Shahmohamadloo^*1^, Xavier Ortiz^2,3^, Claire Holeton^2^, Richard Chong-Kit^2^, David G. Poirier^2^, Satyendra P. Bhavsar^2,4^, and Paul K. Sibley^1^

^1^ School of Environmental Sciences, University of Guelph, Guelph, Ontario, Canada

^2^ Ministry of the Environment, Conservation and Parks, Toronto, Ontario, Canada

^3^ School of Environmental Studies, Queen’s University, Kingston, Ontario, Canada

^4^ Department of Physical & Environmental Sciences, University of Toronto, Toronto, Ontario, Canada

*Corresponding Author: rshahmoh@uoguelph.ca

**TABLES**

**Table S1.** Recipe for BG-11 Trace Metals Solution

| Component | Amount | Stock Solution Concentration | Final Concentration |
| --- | --- | --- | --- |
| H_3_BO_3_ | 2.86 g L^-1^ | - | 46 mM |
| MnCl_2_·4H_2_O | 1.81 g L^-1^ | - | 9 mM |
| ZnSO_4_·7H_2_O | 0.22 g L^-1^ | - | 0.77 mM |
| Na_2_MoO_4_·2H_2_O | 0.39 g L^-1^ | - | 1.6 mM |
| CuSO_4_·5H_2_O | 0.079 g L^-1^ | - | 0.3 mM |
| Co(NO_3_)_2_·6H_2_O | 49.4 mg L^-1^ | - | 0.17 mM |

**Table S2.** Recipe for BG-11 Vitamin B_12_ + Biotin Solution

| Component | Amount | Stock Solution Concentration | Final Concentration |
| --- | --- | --- | --- |
| Vitamin B_12_ | 0.001 mL | 0.05 mL/50mL dH_2_O | - |
| Biotin | 0.01 mL | 0.5 mL/50mL dH_2_O | - |

**Table S3.** Recipe for BG-11 liquid media

| Component | Amount | Stock Solution Concentration | Final Concentration |
| --- | --- | --- | --- |
| NaNO_3_ | 10 mL L^-1^ | 30.0 g/200 mL dH_2_0 | 17.6 mM |
| K_2_HPO4 | 10 mL L^-1^ | 0.80 g/200 mL dH_2_0 | 0.23 mM |
| MGSO_4_·7H_2_O | 10 mL L^-1^ | 1.50 g/200 mL dH_2_0 | 0.3 mM |
| CaCl_2_·2H_2_O | 10 mL L^-1^ | 0.72 g/200 mL dH_2_0 | 0.24 mM |
| Citric Acid·H_2_O | 10 mL L^-1^ | 0.12 g/200 mL dH_2_0 | 0.031 mM |
| Ferric Ammonium Citrate | 10 mL L^-1^ | 0.12 g/200 mL dH_2_0 | 0.021 mM |
| Na_2_EDTA·2H_2_O | 10 mL L^-1^ | 0.02 g/200 mL dH_2_0 | 0.0027 mM |
| Na_2_CO_3_ | 10 mL L^-1^ | 0.40 g/200 mL dH_2_0 | 0.19 mM |
| BG-11 Trace Metals Solution | 1 mL L^-1^ | - | - |
| Vitamin B_12_ + Biotin Solution | 1 mL L^-1^ | - | - |

**Table S4.** Physical and chemical composition of BG-11 medium at start of the experiment

| Properties | Value | Units |
| --- | --- | --- |
| pH | 7.38 | None |
| Conductivity | 2270 | uS cm^-1^ |
| Alkalinity; total fixed endpoint | 26.2 | mg L^-1^ CaCO_3_ |
| Anions | 21.0 | meq L^-1^ |
| Chloride | 24.6 | mg L^-1^ |
| Aluminum | 0.08 | mg L^-1^ |
| Barium | 0.004 | mg L^-1^ |
| Berylium | 0.004 | mg L^-1^ |
| Cadmium | 0.004 | mg L^-1^ |
| Calcium | 9.49 | mg L^-1^ |
| Chromium | 0.008 | mg L^-1^ |
| Cobalt | 0.008 | mg L^-1^ |
| Copper | 0.016 | mg L^-1^ |
| Iron | 1.16 | mg L^-1^ |
| Lead | 0.02 | mg L^-1^ |
| Magnesium | 6.93 | mg L^-1^ |
| Manganese | 0.424 | mg L^-1^ |
| Molybdenum | 0.120 | mg L^-1^ |
| Nickel | 0.04 | mg L^-1^ |
| Potassium | 18.2 | mg L^-1^ |
| Silver | 0.02 | mg L^-1^ |
| Sodium | 428.0 | mg L^-1^ |
| Strontium | 0.004 | mg L^-1^ |
| Titanium | 0.004 | mg L^-1^ |
| Vanadium | 0.004 | mg L^-1^ |
| Zinc | 0.0044 | mg L^-1^ |
| Sulphate | 950.0 | mg L^-1^ |
| Nitrogen; ammonia + ammonium | 0.351 | mg L^-1^ |
| Nitrogen; nitrite | 0.008 | mg L^-1^ |
| Nitrogen; nitrate + nitrite | 270.0 | mg L^-1^ |
| Phosphorus; phosphate | 7.67 | mg L^-1^ |
| Carbon; dissolved organic | 3.6 | mg L^-1^ |
| Carbon; dissolved inorganic | 3.3 | mg L^-1^ |
| Silicon; reactive silicate | 2.02 | mg L^-1^ |
| Nitrogen; total | 274.0 | mg L^-1^ |
| Phosphorus; total | 7.7 | mg L^-1^ |

**Table S5.** Application of first order rate kinetics to assess the relationship between the production of microcystin-LR (*μ*_mcyst-LR_) and [D-Asp^7^]-microcystin-LR (*μ*_mcyst-dmLR_) over cell growth (*μ*_g_) in the experimental cultures

| Day | *μ*_mcyst-LR_/*μ*_g_ | Implication for *μ*_mcyst-LR_ | *μ*_mcyst-dmLR_/*μ*_g_ | Implication for *μ*_mcyst-dmLR_ |
| --- | --- | --- | --- | --- |
| 1 | -0.07 | Not occurring | -0.03 | Not occurring |
| 4 | 0.82 | Occurring but not equal to *μ*_g_ | 0.71 | Occurring but not equal to *μ*_g_ |
| 5 | 4.05 | Faster than *μ*_g_ | 3.75 | Faster than *μ*_g_ |
| 6 | 0.33 | Not occurring | 0.31 | Not occurring |
| 7 | 0.06 | Not occurring | 0.15 | Not occurring |
| 8 | 0.68 | Occurring but not equal to *μ*_g_ | 1.83 | Faster than *μ*_g_ |
| 11 | 0.84 | Occurring but not equal to *μ*_g_ | 1.35 | Faster than *μ*_g_ |
| 12 | -1.49 | Not occurring | -1.62 | Not occurring |
| 13 | 0.95 | Almost equal to *μ*_g_ | 1.39 | Faster than *μ*_g_ |
| 15 | 2.06 | Faster than *μ*_g_ | 2.52 | Faster than *μ*_g_ |
| 18 | 0.22 | Not occurring | -0.08 | Not occurring |
| 19 | 0.60 | Occurring but not equal to *μ*_g_ | 0.37 | Not occurring |
| 20 | 1.50 | Faster than *μ*_g_ | 1.55 | Faster than *μ*_g_ |
| 21 | 1.97 | Faster than *μ*_g_ | 2.00 | Faster than *μ*_g_ |
| 25 | 0.92 | Almost equal to *μ*_g_ | 0.96 | Almost equal to *μ*_g_ |
| 26 | 0.32 | Not occurring | 0.27 | Not occurring |
| 28 | 1.72 | Faster than *μ*_g_ | 2.67 | Faster than *μ*_g_ |
| 31 | 2.14 | Faster than *μ*_g_ | 3.90 | Faster than *μ*_g_ |
| 33 | -1.14 | Not occurring | 0.17 | Not occurring |
| 34 | 0.94 | Almost equal to *μ*_g_ | 0.01 | Not occurring |
| 35 | -0.05 | Not occurring | 0.16 | Not occurring |
| 39 | 0.85 | Occurring but not equal to *μ*_g_ | 1.30 | Faster than *μ*_g_ |
| 42 | 0.85 | Occurring but not equal to *μ*_g_ | 0.08 | Not occurring |
| 46 | 1.87 | Faster than *μ*_g_ | 1.52 | Faster than *μ*_g_ |

[Orr *et al*. (2018)](#Orretal2018).

**FIGURES**

**Figure S1.** Structure of microcystin-LR (R = CH_3_) and [D-Asp^7^]-microcystin-LR (R = H).


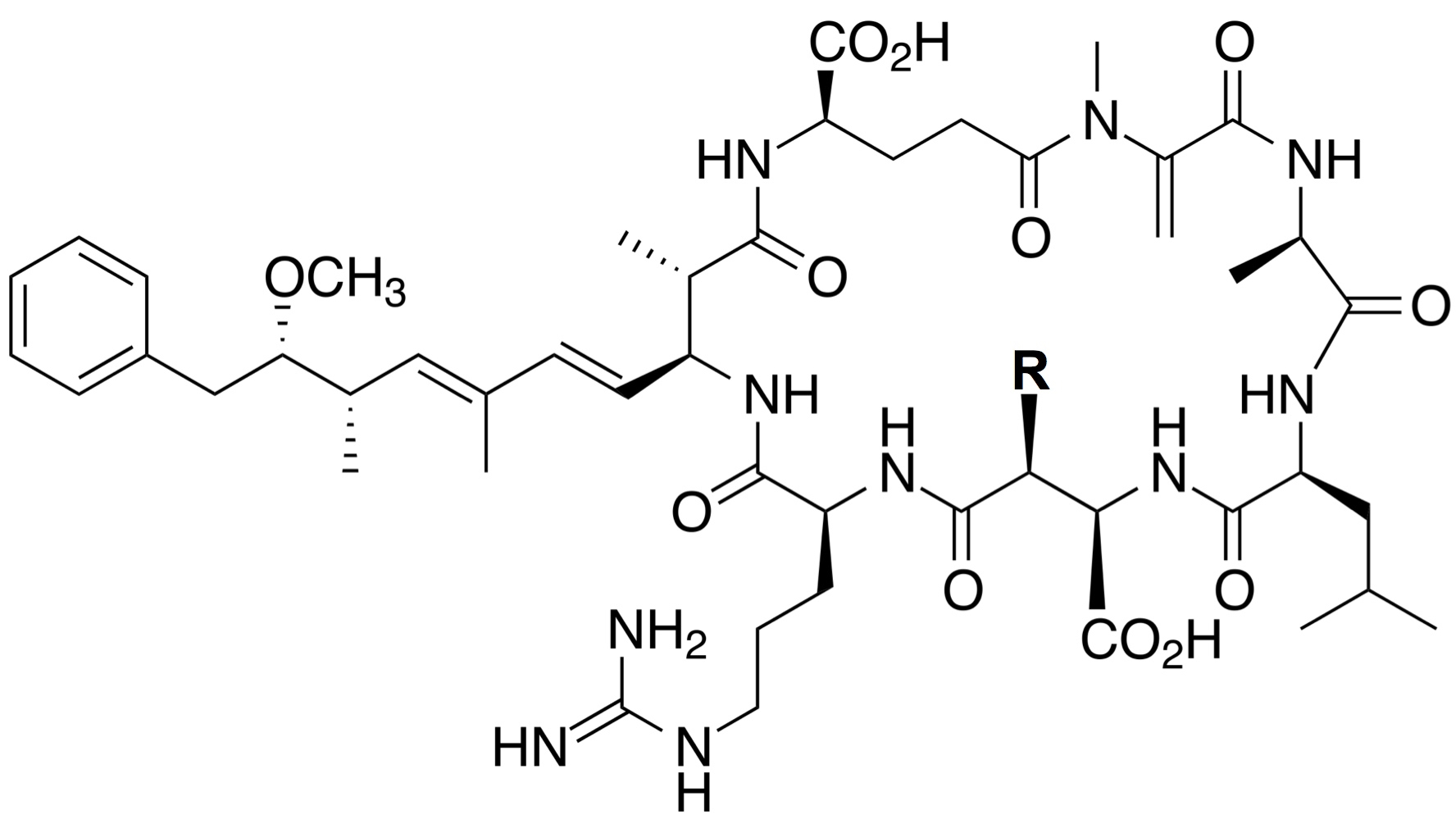

Supplement: Supplementary file 1 [file mmc1.docx]
